# The complete mitochondrial genome of the benthic diatom *Pleurosigma inscriptura*

**DOI:** 10.1080/23802359.2021.1945970

**Published:** 2021-08-09

**Authors:** YuJin Jeong, JunMo Lee

**Affiliations:** Department of Oceanography, Kyungpook National University, Daegu, Korea

**Keywords:** *Pleurosigma inscriptura*, Bacillariophyceae, mitochondrial genome, phylogenetic analysis

## Abstract

*Pleurosigma inscriptura* M. A. Harper 2009 is a marine diatom in Naviculales (Bacillariophyceae) order distributed in New Zealand, South America, Argentina, and Korea. We assembled the complete mitochondrial genome sequence of *Pleurosigma inscriptura* (38,013 bp), and annotated 34 protein-coding genes, 25 transfer RNAs, and 2 ribosomal RNAs. We analyzed a maximum-likelihood tree using conserved 34 mitochondrial genes from Bacillariophyta species. In the mitochondrial phylogeny, *P*. *inscriptura* showed a strong monophyletic relationship with *Haslea nusantara* and *Navicula ramosissima*.

*Pleurosigma inscriptura* M. A. Harper 2009, belongs to order Naviculales (Bacillariophyceae), is a kind of marine pennate diatom which is one of the important primary producers (Nelson et al. 1995; Harper et al. 2009) . *Pleurosigma* lives in a benthic environment, and includes epiphytic species with gliding movement over the surface area (Happey-Wood and Jones 1988; Sterrenburg 1991; Grossi et al. 2004; Al-Handal and Wulff 2008; Poulin et al. 2014). *Pleurosigma* was characterized by sigmoid raphe having a convex valve, and striated areola (Harper et al. 2009; Sar et al. 2012; Al-Handal et al. 2014; Poulin et al.^2014^). The valve outline of *P*. *inscriptura* is lanceolate with a gently sigmoid raphe, and their raphe angle is smaller than other *Pleurosigma* species (Hasle and Syvertsen 1996; Harper et al. 2009). *Pleurosigma inscriptura* is distributed in New Zealand, South America, Argentina, and Korea (Sar et al. 2013; Harper et al. 2012; Lee and Park 2015). Since *P*. *inscriptura* was reported as a novel species (Harper et al. 2009), there is no report about molecular data in public databases (e.g. NCBI). In this study, we analyzed the mitochondrial genome of *P*. *inscriptura* (MW566731) isolated from Gijang, Busan, Korea (35°13′05.9″N, 129°13′47.3″E). The culture strain was deposited at Marine Ecological Genomics Lab. in Kyungpook National University (isolate MEG005; junmolee@knu.ac.kr).

To establish monoclonal strain of the target species, single cell isolation was performed by customized Pasteur pipettes (glass) under light microscope, and cultured under L1 medium (L1 media kit, https://ncma.bigelow.org/MKL150L). The isolate was identified by light microscopic (LM; Nikon ECLIPSE Ts2, Nikon, Tokyo, Japan) and scanning electron microscopic (SEM; Hitachi SU8220; Hitachi Ltd., Tokyo, Japan) observation based on previously described morphological characteristics (Harper et al. 2009; Lee and Park 2015). The morphological characteristics of *P*. *inscriptura* MEG005 are as follows: length of apical axis 104–115 μm (avg. 111 μm, *n* = 100), width of transapical axis 15–25 μm (avg. 21 μm, *n* = 100; Supplementary Figure S1(A)), lanceolate valves, 3.6° raphe angle (Supplementary Figure S1(B)), a small oval central nodule with no hyaline areas (Supplementary Figure S1(C)), 20 striae in 10 μm and small triangle of internal polar raphe ends (Supplementary Figure S1(D)).

**Figure 1.**
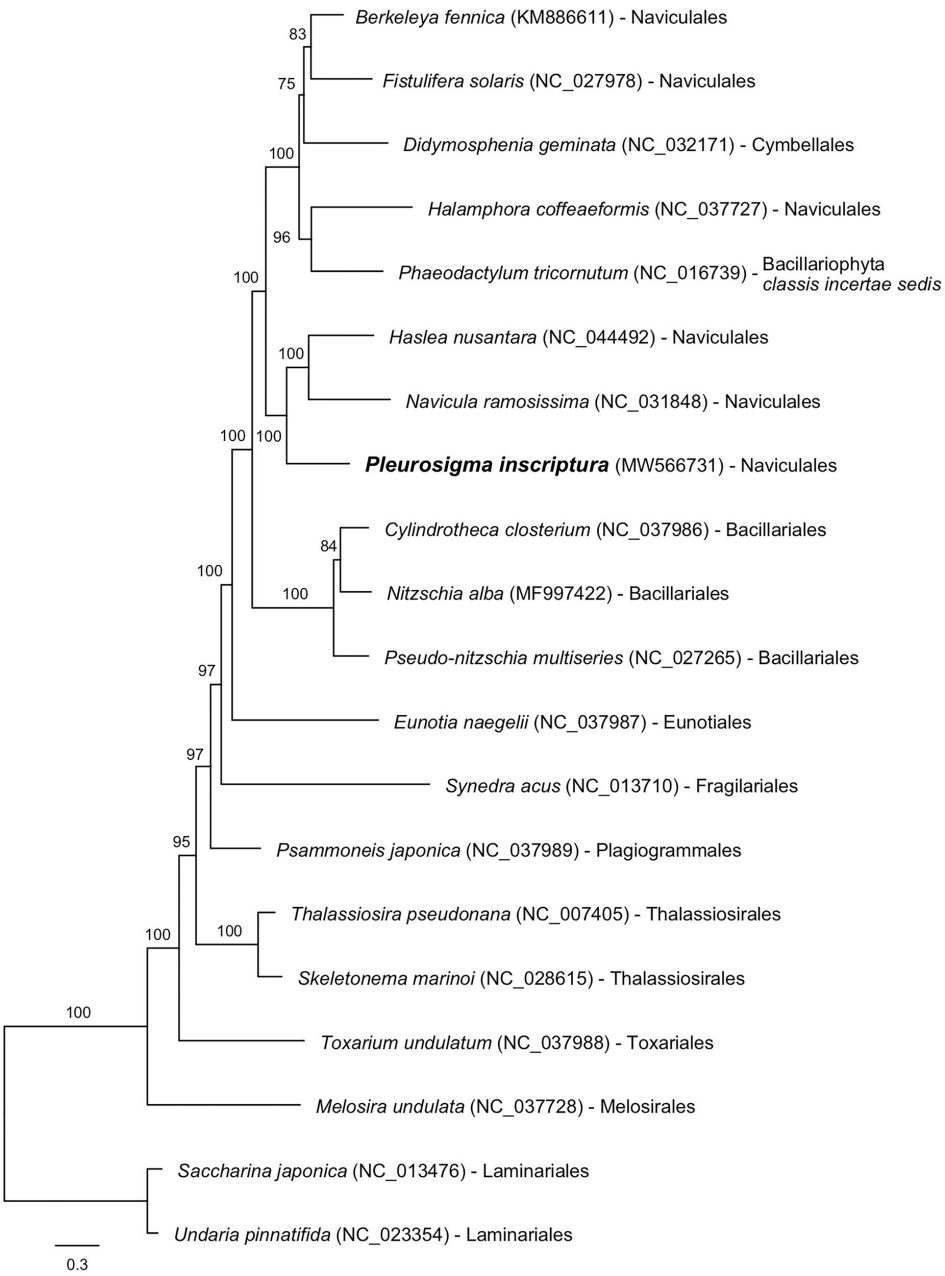
Maximum-likelihood (ML) tree using 34 concatenated mitochondrial proteins from eighteen Bacillariophyta and two Ochrophyta (outgroup) species.

We have done DNA extraction using the DNeasy Plant Mini-DNA kit (Qiagen, Hilden, Germany). Genome sequencing of *P*. *inscriptura* (NCBI BioProject PRJNA717901; SRR14087012, 12.5 Gbp) and their *de novo* genome assembly were conducted by Illumina Novaseq6000 (Illumina, San Diego, CA; 150 bp paired-end library), and SPAdes assembler (v3.14.2; Bankevich et al. 2012), respectively. Mitochondrial genome contigs of *P*. *inscriptura* were sorted by local BLASTn search (*e*-value cutoff = 1.e − 05) with the mitochondrial genome of *Berkeleya fennica* (KM886611) as reference, and re-assembled as a circular mitochondrial genome from the sorted contigs. The circular form of mitochondrial genome (MW566731) was validated using the read-mapping method (bowtie2 v2.4.2; Langmead and Salzberg 2012).

CLC Main Workbench v20.0 (CLC Bio, Aarhus, Denmark) was used to annotate mitochondrial genes that manually predicted by BLASTx search (NCBI nr database; *e*-value cutoff = 1.e − 05) with codon table 4 (The Mold, Protozoan, and Coelenterate Mitochondrial Code). Ribosomal RNAs (rRNAs) were predicted by the RNAmmer 1.2 Server (Lagesen et al. 2007). Transfer RNAs (tRNAs) were analyzed by tRNA scan-SE 2.0 (Lowe and Chan 2016; Chan and Lowe 2019) and ARAGORN programs (Laslett and Canback 2004), respectively. A total 34 protein-coding genes, 25 transfer RNAs, 2 rRNAs is annotated in the mitochondrial genome of *P*. *inscriptura* (MW566731). Intron sequences of protein coding genes are present in *rps*3 (two introns), *nad*2 and *nad*11 (one intron).

To construct the concatenated alignment of mitochondrial genes, conserved 34 protein-coding genes from 18 Bacillariophyta and two outgroup species were used, and each homologous gene set was aligned by MAFFT (v7.313; Katoh and Toh 2008) with default options. The maximum-likelihood (ML) tree using the concatenated alignment was constructed by IQ-tree program (v1.6.12; Nguyen et al. 2015) with the options as follows: the gene partition information (-q), the model test (-m TEST), and ultrafast bootstrapping with 1000 replications (-bb 1000). *Pleurosigma inscriptura* is monophyly with *Haslea nusantara* and *Navicula ramosissima* (bootstrap support 100%; Figure 1), and this clade is clustered with the clade of *Berkeleya fennica, Fistulifera solaris, Didymosphenia geminata, Halamphora coffeaeformis,* and *Phaeodactylum tricornutum* (bootstrap support 100%). In the clade, almost all species belong to Naviculales order but *D*. *geminate* and *P*. *tricornutum* are Cymbellales and Bacillariophyta *classis incertae sedis*, respectively (Guiry and Guiry 2021). In previous studies, *D*. *geminate* and *P*. *tricornutum* show monophyletic relationship with Naviculales order that the phylogenetic tree was constructed by 27 mitochondrial protein-coding genes (Aunins et al. 2018). Therefore, we postulate that *D*. *geminate* and *P*. *tricornutum* could be regarded as the Naviculales order. More mitochondrial genome samples in Naviculales order will provide a better understanding of phylogenetic relationship between genera.

## Data Availability

The data that support the findings of this study are openly available in GenBank at https://www.ncbi.nlm.nih.gov/genbank/, reference number ‘MW566731’. Raw reads of genome sequencing data in this study were uploaded to the NCBI Sequence Read Archive (SRA) database (BioProject PRJNA717901; SRR14087012).
